# Vitamin D prophylaxis in infancy

**DOI:** 10.1186/s12887-021-02776-z

**Published:** 2021-09-08

**Authors:** Sophie Jullien

**Affiliations:** Barcelona Institute for Global Health, University of Barcelona, Barcelona, Spain

**Keywords:** 25-hydroxyvitamin D, Infants, Vitamin D supplementation, Rickets

## Abstract

We looked at existing recommendations and supporting evidence on the effectiveness of vitamin D supplementation in infancy for reducing vitamin D deficiency and for preventing rickets and infections. We also looked at optimal dose of vitamin D and the age until which vitamin D supplementation is beneficial.

We conducted a literature search up to the 17th of July 2019 by using key terms and manual search in selected sources. We summarized the recommendations and the strength of the recommendation when and as reported by the authors. We summarized the main findings of systematic reviews with the certainty of the evidence as reported.

A daily dose of 400 international units of vitamin D in infants has shown to be effective for improving bone health and preventing rickets. Evidence is more robust in groups of infants and children at risk. Vitamin D supplementation is well tolerated, and not associated with toxicity. Higher doses have not shown to add benefit while it could potentially cause toxic blood levels and hypercalcemia. Adequate levels of vitamin D might not be achieved with lower daily doses. Universal vitamin D supplementation starting shortly after birth, regardless of the mode of feeding and until 12 months of age, is strongly recommended. Beyond 12 months of age vitamin D supplementation is recommended only in groups of children at risk.

## Background

### Introduction

The World Health Organization (WHO) European Region is developing a new pocket book for primary health care for children and adolescents in Europe. This article is part of a series of reviews, which aim to summarize the existing recommendations and the most recent evidence on preventive interventions applied to children under 5 years of age to inform the WHO editorial group to make recommendations for health promotion in primary health care. In this article, we looked at existing recommendations and supporting evidence on the effectiveness of vitamin D supplementation in reducing vitamin D deficiency and in preventing rickets and infections.

### Why is vitamin D important?

Vitamin D is essential in the metabolism of calcium and phosphate, skeletal growth and bone health, but is also involved in other functions such as the modulation of the function of activated B and T lymphocytes, insulin production, the secretion of thyroid stimulating hormone and myocardial contractility [1].

### Context

Vitamin D from the diet is limited, the main natural source of vitamin D being from the action of the sunlight on the skin. However, several factors such as cultural practices and sun protection for prevention of cancer has conditioned vitamin D deficiency and rickets to reappear as a global health problem. Low concentration of vitamin D in breast milk, and the recommendation of keeping newborns and infants out of direct sun exposure are all factors that contribute to the risk of vitamin D deficiency in infants [2]. Vitamin D deficiency is usually defined as serum levels of 25-hydroxyvitamin D lower than 50 nmol/l (equivalent to 20 ng/ml), while serum levels superior to 75 nmol/l (equivalent to 30 ng/ml) ensure adequate metabolic demands and health [3].

## Key questions

1. Is vitamin D supplementation in infancy effective in reducing vitamin D deficiency and in preventing rickets and infections? (Table 1)
2. Which dose of vitamin D is more effective and safer in preventing rickets in infancy?
3. Until which age is prophylactic vitamin D beneficial to infants?

**Table 1:** Key questions for vitamin D prophylaxis in infancy

	Key question 1	Key question 2	Key question 3
**Participants**	Infants	Infants	Infants without risk factors Infants with risk factors
**Intervention**	Vitamin D supplement at any dose	Dose A of vitamin D	Vitamin D supplement until 12 months of age
**Comparison**	Placebo or no vitamin D supplement	Dose B of vitamin D	Vitamin D supplement until more than 12 months of age
**Outcomes**	Clinical outcomes: rickets (clinical or radiological signs), infections Anthropometric outcomes such as mineral bone density Levels of serum vitamin D (25OHD)		
**Adverse effects**	Any adverse effect		

## Search methods and selected manuscripts

We described the search methods, data collection and data synthesis in the second paper of this supplement [4].

The search was conducted on the 17th of July 2019, using the search term “vitamin D”. Vitamin D supplementation in infants is addressed by WHO and provided by the e-Library of Evidence for Nutrition Actions (eLENA). The search on the US Preventive Services Task Force (USPSTF) returned five entries, but none of them included the paediatric population, and vitamin D in children was not identified as a recommendation in progress. From the PrevInfad workgroup (Spanish Association of Primary Care Pediatrics), recommendations were found for Vitamin D prophylaxis during infancy and early childhood, although dated from August 2009. Two guidelines were identified from the National Institute for Health and Care Excellence (NICE).

The search in the Cochrane library using the date of publication from 2010 as filter yielded 63 reviews and 12 protocols. By screening the titles and abstracts, we included two systematic reviews. Twelve additional reviews were published before 2010 and we identified one relevant systematic review from 2007. We identified three protocols from which the findings of the corresponding systematics reviews would be relevant for this article. We contacted the authors of three protocols; all of them replied that their systematic review was expected to be published in the coming months. We updated the search on the 10th of October 2019, none of them were published in the Cochrane library, but we had access to the unpublished findings of the review by Huey et al.

We identified two additional studies by hand search of the references of included papers. One paper on global consensus recommendations on prevention and management of nutritional rickets, and one systematic review gathering the evidence on vitamin D requirements in infancy from trials published between 2009 and 2016.

All the included manuscripts for revision in this article are displayed in Table 2.

**Table 2:** Included manuscripts for revision

Sources	Final selected manuscripts
**WHO**	• eLENA statement (recommendations) [5]
**USPSTF**	None
**PrevInfad**	• 2009 recommendations and supporting document [6]
**CDC**	• Recommendations [7]
**NICE**	• Vitamin D: supplement use in specific population groups; 2014; updated in 2017 (Guideline) [8]
	• Maternal and child nutrition; 2008, updated in 2014 (Guideline) [9] (Note: recommendations on vitamin D in this guideline have been replaced by the Vitamin D 2014 guideline)
**Cochrane Library**	• Yakoob 2016 – Vitamin D supplementation for preventing infections in children under five years of age (Systematic review) [10]
	• Winzenberg 2010 – Vitamin D supplementation for improving bone mineral density in children (Systematic review) [11]
	• Lerch 2007 – Interventions for the prevention of nutritional rickets in term born children [12]
	• Yu 2017 – The effects of oral vitamin D supplementation on linear growth and non-communicable diseases among infants and children younger than five years of age (Protocol) [13]. Access to the unpublished stage of the systematic review: Huey 2019 [14]
	• Tan 2018 – Vitamin D supplementation for term breastfed infants to prevent vitamin D deficiency and improve bone health (Protocol) [15]
	• Pharande 2015 – Vitamin D supplementation for prevention of vitamin D deficiency in preterm and low birth weight infants (Protocol) [16]
**Others**	• Munns 2016 – Global consensus recommendations on prevention and management of nutritional rickets (Recommendations and supporting evidence from literature review) [17]
	• Mimouni 2017 – Vitamin D requirements in infancy: a systematic review (Systematic review) [18]

*Abbreviations***:***CDC* Centers for Disease Control and Prevention, *NICE* National Institute for Health and Care Excellence, *PrevInfad* PrevInfad workgroup from the Spanish Association of Primary Care Pediatrics, *USPSTF* US Preventive Services Task Force, *WHO* World Health Organization

## Existing recommendations

We summarized the existing recommendations and the strength of recommendations as per their authors in Table 3.

**Table 3:** Summary of existing recommendations

Sources	Ref	Date	General recommendations for use of prophylactic vitamin D in infants
**WHO**	[5]	2019	“Current evidence suggests that vitamin D supplements may be effective in preventing rickets, particularly for infants and children who may be at higher risk due to limited sun exposure or those with darker skin pigmentation, however further research is needed before specific recommendations can be made.” (*Category 2 intervention*)^a^
**PrevInfad**	[6]	2009	Breastfed infants under one year of age have to receive vitamin D supplement of 400 IU/day, starting from the first days of life, and until the child is fed with one litre per day of formula milk fortified with vitamin D. (*Grade B recommendation*) All infants under one year of age fed with substitute of human milk with less than one litre per day of formula have to receive vitamin D supplement of 400 IU/day. (*Grade B recommendation*) Children or adolescents with any risk factor^b^ of vitamin D deficiency and who do not acquire 400 IU/day of vitamin D with one litre of fortified milk or enriched aliments (one portion of cereals and one yolk contains 40 IU of vitamin D each) or adequate solar exposition have to receive vitamin D supplement of 400 IU/day. (*Grade B recommendation*) For children older than one year or adolescents, daily solar exposition at midday without protection during 10 to 15 min in spring, summer and fall is generally recommended for an adequate production of vitamin D. In winter, vitamin D is not produced above 42° North latitude. (*Grade I recommendation*) Premature children under one year of corrected age need vitamin D intake of 200 IU/kg/day until a maximum of 400 IU/day. (*Grade A recommendation*)
**CDC**	[7]	Updated 2018	“All children need vitamin D beginning shortly after birth. • Children younger than 12 months old need 400 IU of vitamin D each day. • Children 12 to 24 months old need 600 IU of vitamin D each day.” “Breast milk usually does not provide all the vitamin D a baby needs, so breastfed babies will need a supplemental source […].” “32 oz of standard infant formula per day contains 400 IU of vitamin D. If your baby is drinking less than this amount per day, he or she may need a vitamin D supplement […].”
**NICE**	[9]	2008	“GPs and health visitors should offer children’s Healthy Start vitamin supplements (vitamins A, C and D) to all children aged from 6 months to 4 years in families receiving the Healthy Start benefit.”
**Global consensus**	[17]	2016	“Vitamin D supplementation for the prevention of rickets and osteomalacia • 400 IU/d (10 μg) is adequate to prevent rickets and is recommended for all infants from birth to 12 months of age, independent of their mode of feeding. (1 ⊕ ⊕⊕)^c^ • Beyond 12 months of age, all children and adults need to meet their nutritional requirement for vitamin D through diet and/or supplementation, which is at least 600 IU/d (15 μg), as recommended by the Institute of Medicine. (1 ⊕ ⊕⊕)^c^” “Candidates for preventive vitamin D supplementation beyond 12 months of age In the absence of food fortification, vitamin D supplementation should be given to: • Children with a history of symptomatic vitamin D deficiency requiring treatment. (1 ⊕ ⊕⊕)^c^ • Children and adults at high risk of vitamin D deficiency, with factors or conditions that reduce synthesis or intake of vitamin D. (1 ⊕ ⊕⊕)^c^” “Prevention of nutritional rickets/osteomalacia: identification of risk factors • In addition to an intake of 400 IU/d of vitamin D, complementary foods introduced no later than 26 weeks should include sources rich in calcium. (1 ⊕ ⊕⊕)^c^ • An intake of at least 500 mg/d of elemental calcium must be ensured during childhood and adolescence. (1 ⊕ ⊕⊕)^c^” “Public health strategies for rickets prevention: • Universally supplement all infants with vitamin D from birth to 12 months of age, independent of their mode of feeding. Beyond 12 months, supplement all groups at risk and pregnant women. Vitamin D supplements should be incorporated into childhood primary health care programs along with other essential micronutrients and immunizations (1 ⊕ ⊕⊕)^c^, and into antenatal care programs along with other recommended micronutrients. (2 ⊕ ⊕○)^c^”

*Abbreviations***:***CDC* Centers for Disease Control and Prevention, *NICE* National Institute for Health and Care Excellence, *PrevInfad* PrevInfad workgroup from the Spanish Association of Primary Care Pediatrics, *USPSTF* US Preventive Services Task Force, *WHO* World Health Organization

^a^The eLENA groups the interventions into three categories according to the availability of the evidence as follows. Category 1: Interventions for which there are guidelines that have been recently approved by the WHO Guidelines Review Committee. Category 1 interventions also include those supported by recommendations and other forms of guidance that have been adopted or endorsed by the World Health Assembly. Category 2: Interventions for which systematic review(s) have been conducted but no recent guidelines are yet available that have been approved by the WHO Guidelines Review Committee. Category 3: Interventions for which available evidence is limited and systematic reviews have not yet been conducted

^b^See paragraph below on ‘Risk factors for vitamin D deficiency’

^c^Recommendations were graded as 1 for strong recommendation or 2 for weak recommendation; and quality of evidence was assessed as ⊕ ⊕ ⊕ high quality (prospective cohort studies or randomized controlled trials at low risk of bias), ⊕ ⊕ ○ for moderate quality (observational studies or trials with methodological flaws, inconsistent or indirect evidence), and ⊕○○ for low quality (case series or non-systematic clinical observations) [17]

The Global consensus recommendations were established by the initiation of the European Society for Pediatric Endocrinology, based on evidence up to 2014 [17]. The expert panel included members from the Pediatric Endocrine Society, the Asia Pacific Pediatric Endocrine Society, the Japanese Society for Pediatric Endocrinology, the Sociedad Latino-Americana de Endocrinología Pediátrica, the Australasian Pediatric Endocrine Group, the Indian Society for Pediatric and Adolescent Endocrinology, the African Society for Pediatric and Adolescent Endocrinology, the Chinese Society of Pediatric Endocrinology and Metabolism, the British Nutrition Society, and the European Society for Pediatric Gastroenterology Hepatology and Nutrition.

### Risk factors for vitamin D deficiency

Most recommendations refer to groups of infants and children at high risk for vitamin D deficiency. Identified risk factors for vitamin D deficiency are [6, 17, 19]:

- In newborns and small infants:
  - o Maternal vitamin D deficiency: mothers with restricted sun exposure, with dark skin colour, who wear veil, multiparous, with low vitamin D intake
  - o Prolonged exclusive breastfeeding without vitamin D supplementation
  - o Preterm babies and small for gestational age
- In older infants and children:
  - o Decrease in vitamin D synthesis from restricted sun exposure: little time outdoors, use of protection factor > 8 (inhibits synthesis > 95%), dark skin colour, cultural practices, veils, clothes, crystal, etc.
  - o Decrease in vitamin D intake: prolonged exclusive breastfeeding without supplementation, poor nutrition, low intake of foods containing vitamin D
  - o Certain medical conditions and chronic diseases:
    - ▪ Intestinal malabsorption: small bowel disorders (such as coeliac disease), pancreatic insufficiency (such as cystic fibrosis), biliary obstruction (such as biliary atresia)
    - ▪ Reduced synthesis or increased degradation of 25-(OH) D or 1,25(OH)_2_D: chronic liver or renal diseases, treatment with rifampicin, isoniazid and anticonvulsants

## Existing evidence

Three Cochrane reviews assessed the effectiveness of vitamin D supplements for improving bone health [11], on linear growth and non-communicable diseases [14], and for preventing infections [10] in children. An older Cochrane review looked at the effectiveness of vitamin D supplements for preventing rickets [12]. Although published in 2007, we included this older review in this summary as it constitutes part of the supporting evidence for current recommendations [5, 6].

Five studies were identified by the global consensus panel and served as reference for developing their recommendations on vitamin D supplementation for the prevention of rickets and osteomalacia [17]. Among them, one study (Beser 1994) was included in the Lerch 2007 Cochrane review, two studies [20, 21] were not included but published prior to the publication of the identified Cochrane systematic reviews for this summary, and two studies were published afterwards [22, 23]. The aim of the Mimouni 2017 systematic review was to review more recent publications (between 2009 and 2016) for new evidence related to vitamin D dosages in healthy infants [18].

### Vitamin D supplementation for preventing rickets and improving bone health

The Lerch 2007 Cochrane review looked at the effects of several interventions (vitamin D supplementation, vitamin D and calcium supplementation, or increased sun exposure) for preventing nutritional rickets in term born children [12]. Four trials were identified and included children aged between 9 months and 2 years from China, France and Turkey. In the trial conducted in Turkey, none of the 300 children who received 400 international units (IU) per day of vitamin D developed rickets versus 14 children out of 372 from the control group (relative risk [RR] 0.04; 95% confidence interval [CI] 0 to 0.71; one trial). In one trial conducted in China, authors compared a combined intervention of vitamin D supplementation, calcium and nutritional counselling against no intervention. Despite low compliance in the intervention group, 100/183 children and 33/46 children developed signs of nutritional rickets in the intervention and control groups, respectively (RR 0.76; 95% CI 0.61 to 0.95; one trial). In two trials conducted in China and France, no children developed rickets in both intervention and control groups.

In addition to the Turkish study already included in Lerch 2007, two studies were identified by the general consensus panel that looked at the effect of vitamin D supplementation for preventing rickets [17]. A randomized controlled trial (RCT) conducted in Chinese children showed that vitamin D supplementation of 100, 200 or 400 IU per day prevented radiographic signs of rickets at 6 months of age [21]. Similarly, two-year surveillance data from Canada showed that there was no case of radiographically confirmed rickets among infants receiving regular vitamin D supplementation of 400 IU per day from birth [22].

As there is limited evidence from studies looking at radiological or clinical signs of rickets as outcome, we included studies that looked at other related outcomes such as levels of 25OHD and bone mineral density. The Winzenberg 2010 Cochrane review looked at the effectiveness of vitamin D supplementation for at least 3 months versus placebo in healthy children aged from 1 month to < 20 years for improving bone mineral density [11]. Six RCTs (541 children receiving vitamin D and 343 children receiving placebo) were included for meta-analysis. These studies included children aged between eight and 17 years from Hong Kong, Finland, Pakistan and Lebanon. With a follow-up of one to 2 years, vitamin D had no effect on total body bone mineral density (standardized mean difference [SMD] 0.1 [95%CI − 0.06 to 0.26]; five studies, 672 participants; high certainty evidence) or lumbar spinal bone mineral density (SMD 0.15 [95%CI − 0.01 to 0.31]; five studies, 660 participants; high certainty evidence), and probably had no effect on hip bone mineral density (SMD 0.06 [95% CI − 0.18 to 0.29]; four studies, 639 participants; moderate certainty evidence) and forearm bone mineral density (SMD 0.04 [95%CI − 0.36 to 0.45]; three studies, 427 participants; moderate certainty evidence).

### Vitamin D supplementation and linear growth

The Huey 2019 Cochrane review (yet unpublished) evaluated the effects of vitamin D supplementation on linear growth [14]. For all the outcomes addressed by this review, 60 RCTs or quasi-RCTs were included. Most of them were conducted in the US, India, Finland, Canada, and Iran. The duration of follow-up for the different interventions ranged from a few days to 24 months. The main findings were as follows.

- Vitamin D supplementation in children was not associated with any significant differences in linear growth (mean length/height, in cm) at the end of the supplementation period or at long-term follow-up: vitamin D versus placebo (three RCTs, 120 participants; low certainty evidence), higher-dose versus lower-dose vitamin D (eight RCTs, 240 participants; very low certainty evidence), or vitamin D with micronutrients versus micronutrients alone (one RCT, 25 participants; moderate certainty evidence).
- Vitamin D supplementation in children was not associated with any significant difference in height-for-age Z scores (mean HAZ scores) when compared to placebo (one RCT, 1258 participants; high certainty evidence) or to lower-dose vitamin D (two RCTs, 24 participants; very low certainty evidence).
- Vitamin D supplementation in children compared to placebo was not associated with any significant difference in the prevalence of stunting (one RCT, 1282 participants; high certainty evidence).

### Vitamin D supplementation and other non-communicable diseases outcomes

The yet unpublished Cochrane review aimed to evaluate the effect of vitamin D supplementation in children on atopic diseases (this is asthma, recurring wheeze, dermatitis, and/or rhinitis) and other non-communicable diseases (any type of cancer, type 1 and type 2 diabetes mellitus, insulin resistance, and other autoimmune disorders) as secondary outcomes [14]. They found no significant differences on atopic diseases, and they found no studies assessing other non-communicable diseases.

### Vitamin D supplementation for preventing infections

The Yakoob 2016 Cochrane review looked at the effectiveness of vitamin D supplementation versus placebo or no intervention in children under 5 years of age for preventing infections from RCTs [10]. There was probably no benefit of vitamin D supplementation on the incidence of radiologically confirmed pneumonia (RR 1.06 [95%CI 0.89 to 1.26]; two trials, 3134 participants; moderate certainty evidence) or diarrhoea (no effect, however meta-analysis was not possible for this outcome; two trials, 3134 participants). From one trial (3046 participants), vitamin D supplementation may not have effect on all-cause mortality (RR 1.43 (95%CI 0.54 to 3.74); low certainty evidence) or on cause-specific mortality (RR 1.50 [95%CI 0.42 to 5.30]; low certainty evidence).

### Different dosages of vitamin D supplementation for preventing rickets

Three studies identified by Munns et al. [17] compared the effect of different dosages of vitamin D supplementation [20, 21, 23]. In their consensus statement, the expert panel added that “Among infants and toddlers with 25OHD levels < 50 nmol/L for whom daily vitamin D supplementation may not be ideal, intermittent bolus doses of 50 to 100 000 IU every 3 months hold promise, although a comprehensive understanding of the safety and efficacy of this approach remains to be studied” [17]. The Mimouni 2017 review [18] identified 11 manuscripts from nine RCTs looking at the effect of different doses of vitamin D supplementation in healthy infants [23–33], including one of the three studies identified by Munns et al. [23]. Authors concluded that “there is no additional evidence that larger, more generous amounts of daily vitamin D beyond the recommended 400 IU daily dose, affects any long-term significant outcome. It even appears that larger amount may lead to serum 25(OH) D concentrations that have been reported to be potentially associated with adverse effects.” The basic characteristics and main findings of the 13 studies identified by these two reviews are summarized in Table 4.

**Table 4:** Characteristics and main findings of studies looking at different doses of vitamin D supplementation [17, 18]

Ref	Setting, date of publication	Participants	Interventions: regimens of vitamin D	Findings
[21]	China 1992	312 healthy term infants	(A) 100 IU/day (B) 200 IU/day (C) 400 IU/day Initiated at 3 to 5 days of life, up to 6 months of age.	Adequate levels of 25OHD above the rachitic range (equivalent to severe deficiency of less than 11 ng/mL) were more frequently achieved among infants receiving the 400 IU dosages compared to the 100 and 200 IU dosages.
[20]	Afghanistan 1994	Infants with vitamin D deficiency (25OHD < 25 nmol/L)	(A) Single dose of 600,000 IU at birth (B) Single dose of 200,000 IU at birth (C) 100,000 IU at birth, 3 and 6 months	(C) provided the best protection against vitamin D deficiency without reaching unacceptably high concentrations of 25OHD.
[24]	Louisiana 2010	80 breastfed babies	(A) 200 IU from day 1 to 6 months (B) 200 IU from month 2 to 6 months (C) placebo	No differences in 25OHD, Calcium, or Phosphorous concentrations between (A) and (B) at 2, 4, and 6 months of age. At 4 months of age, the 25OHD serum concentration of (C) was significantly lower than (A) and (B). No cases of rickets in any of the 3 groups. Author conclusion “universal supplementation is not necessary for rickets prevention in Southern Louisiana”
[26]	Iran 2010	129 infants	(A) 200 IU/day (B) 400 IU/day (C) 50,000 IU/month	Serum 25OHD concentration at 6 months of age: (A) 20 to 51 ng/ml (B) 23 to 64 ng/ml (C) 28 to 102 ng/ml
[27]	Spain 2011	88 term infants, not all exclusively breastfed	(A) 400 IU/day for 12 months (B) No vitamin D supplements	Serum 25OHD concentration: • At 3 months of age: (A) 41.8 +/− 16.7 ng/ml; (B) 27.6 +/− 12.8 ng/ml; *p* < 0.001 • At 6 months of age: (A) 43.8 +/− 13.8 ng/ml; (B) 32.5 +/− 8.9 ng/ml; p < 0.001 • At 12 months of age: no differences between (A) and (B) Parathyroid hormone: no differences between (A) and (B) at any time. No clinical rickets in neither group.
[28]	Germany 2011	40 term infants	(A) 250 IU/day (B) 500 IU/day Starting in summer or in winter, follow-up of 6 weeks.	Serum 25OHD concentrations at six weeks: (A) 45.6 ng/ml to 65.6 ng/ml (B) 50 ng/ml to 70 ng/ml No seasonal variations in 25OHD concentrations.
[29]	Finland 2012	113 breastfed infants recruited at 2 weeks of age	(A) 400 IU/day (B) 1200 IU/day (C) 1600 IU/day Follow-up of 12 weeks.	Only (C) maintained serum 25OHD concentration > 32.5 ng/mL in all infants without hypercalcemia or hypercalciuria. In (B) and (C) some infants reached 25OHD concentrations > 80 ng/mL. Bone density (peripheral quantitative computed tomography): no significant differences between the 3 groups.
[30]	Turkey 2013	169 breastfed term babies	(A) 200 IU/day (B) 400 IU/day	Serum 25OHD concentrations at 4 months: (A) median of 39.6 ng/ml (range 17.05 to 106) (B) median of 56.5 ng/ml (range 32 to 150.2); with significant differences (*p* < 0.001) Proportion of infants with serum 25OHD < 30 mg/ml at 4 months: (A) 21.3% (B) 0% No clinical rickets in either group.
[31]	Canada 2013	51 term infants	(A) Vit D_2_ 400 IU/day (B) Vit D_3_ 400 IU/day	No differences in increase of plasma 25OHD from baseline between (A) and (B) after 3 months
[23]	Canada 2013	132 breastfed infants recruited at 1 month of age	(A) 400 IU/day (B) 800 IU/day (C) 1200 IU/day (D) 1600 IU/day Follow-up for 11 months, reassessed at 1 year of age.	Plasma 25OHD levels of ≥50 nmol/L (20 ng/ml) in 97% (95% CI 94 to 100) of infants at 3 months, and sustained in 98% (95% CI 94 to 100) of infants at 12 months in all groups. Plasma 25OHD concentration ≥ 75 nmol/L (30 ng/ml) in 97.5% of infants at 3 months only in (D). The (D) dose led to 25OHD concentration (> 150 ng/ml) that may cause hypercalcemia (potential toxicity). No differences in growth and bone mineral content between groups. Authors concluded that “dosages of vitamin D higher than 400 IU per day provide no additional benefits for bone mineral accretion.”
[32]	Canada 2016	Follow-up of [23] at 3 years of age	Same as above	Bone health at 3 years of age: “Lumbar spine vertebrae 1–4 bone mineral density, lumbar spine and whole body bone mineral content, and mineral accretion, measured by dual-energy X-ray absorptiometry were similar among all treatment groups.”
[33]	Canada 2017	Follow-up of [23] at 3 years of age	Same as above	Body composition (by anthropometric measurements and from the dual-energy X-ray absorptiometry measurements): no differences between the 3 groups. “However when all infants were combined, there was a weak correlation between 25OHD measurements and lean body mass, although this correlation may have only reflected subtle differences in outdoors physical activity rather than differences because of treatment group allocation.”
[25]	Afghanistan 2013	3046 children between 1 and 11 months of age	Vitamin D: (A) 100,000 IU/3 months; 6 doses (B) Placebo/3 months; 6 doses	Occurrence of diarrheic episodes during the 18-months follow-up period: no significant differences between (A) and (B). Serum 25OHD concentrations: not assessed.

### Duration of administration of vitamin D supplementation

While most of the sources recommend vitamin D supplementation in all infants for the first 12 months of age, there is a lack of strong evidence supporting this duration of administration of 12 months. Assessment of risk factors including the overall vitamin D intake through milk and foods containing vitamin D and sun exposure seem key factors leading to the established recommendations.

### Adverse effects

Vitamin D supplementation was well tolerated according to the trials included in Winzenberg 2010 [11]. None of the included trials in Yakoob 2016 reported any adverse effect of vitamin D supplementation, although one infant was found to have high concentration of vitamin D in one trial with no clinical repercussion, and two children had toxic concentrations of vitamin D in another included trial [10].

The recent, yet unpublished Cochrane review evaluated adverse events of oral vitamin D and found that there were no significant differences in the risk of hypercalciuria, hypercalcemia, nor hyperphosphatemia between oral vitamin D and either placebo, lower doses of vitamin D or micronutrients [14].

## Summary of findings

- All infants should receive vitamin D for improving bone health and preventing rickets, starting shortly after birth, regardless of the mode of feeding. Evidence is more robust to support this recommendation in groups of infants and children at risk.
- There is probably no benefit of universal vitamin D supplementation in infants on the incidence of radiologically confirmed pneumonia or diarrhoea.
- A daily dose of 400 international units of vitamin D in infants has shown to be effective for preventing rickets. It is well tolerated, and not associated with toxicity. Higher doses have not shown to add benefit while it could potentially cause toxic blood levels and hypercalcemia. Adequate levels of vitamin D might not be achieved with lower daily doses.
- Universal vitamin D supplementation until 12 months of age is strongly recommended. Beyond 12 months of age vitamin D supplementation is recommended only in groups of children at risk. There is however a lack of evidence supporting this cut off of 12 months, so this age cut off seems arbitrary.

## Data Availability

Not applicable.
